# Long-Term Outcomes After High-Dose-Rate Brachytherapy and Hypofractionated External Beam Radiotherapy in Very High-Risk Prostate Cancer: A 24-Year Follow-Up

**DOI:** 10.3390/biomedicines13061310

**Published:** 2025-05-27

**Authors:** Pedro J. Prada Gómez, Ana L. Rivero Pérez, Joaquín Carballido Rodríguez, Javier Anchuelo Latorre, Rosa Fabregat Borrás, Marina Gutiérrez Ruiz, Cristina Rodríguez-Acosta Caballero, Carlos F. Carrascal Gordillo, Maria P. Galdós Barroso, Paola A. Navarrete Solano

**Affiliations:** 1 Radiation Oncology Department, Hospital Universitario Marqués de Valdecilla, 39008 Santander, Spainpaolaandrea.navarrete@scsalud.es (P.A.N.S.); 2 Radiation Oncology Department, Hospital Universitario Puerta de Hierro, 28222 Madrid, Spain; 3 Radiation Physics Department, Hospital Universitario Marqués de Valdecilla, 39008 Santander, Spain; 4 Radiation Oncology Department, Hospital Universitario Central de Asturias, 33011 Oviedo, Spain; carrascal1128@hotmail.com

**Keywords:** brachytherapy, very high risk, prostate cancer, external beam radiotherapy

## Abstract

**Purpose**: To evaluate the long-term oncological outcomes and toxicity profile based on 24 years of follow-up in patients with localized very high-risk prostate cancer (VHR PCa) treated with a combination of high-dose-rate brachytherapy (HDR-BT) and pelvic external beam radiation therapy (EBRT). **Methods**: A retrospective analysis was conducted on 87 patients with VHR PCa, classified according to National Comprehensive Cancer Network (NCCN) criteria, who received HDR-BT and EBRT. Androgen deprivation therapy (ADT) was administered to 72 patients (82.8%). The primary endpoints were biochemical control and cancer-specific survival (CSS), while the secondary endpoints included local control rates, tumor-free survival (TFS), overall survival (OS), and treatment-related toxicity. **Results**: The 24-year biochemical control rate was 68% (standard deviation [SD]: ±4%), while CSS and TFS at 24 years were 82% (SD ±4%) and 78% (SD ±4%), respectively. Local control rates remained at 98% at 24 years. Furthermore, the OS rate at 24 years was 30%. Multivariate Cox regression analysis identified the T category in the TNM classification as the only factor significantly associated with biochemical control, with 24-year rates of 69%, 71%, and 50% for patients with T-classifications of ≤T2c, T3a, and T3b-T4, respectively (*p* = 0.024). Notably, no grade ≥3 late toxicities were observed during the follow-up period. **Conclusions**: The 24-year outcomes support the viability and therapeutic efficacy of EBRT combined with a conformal HDR-BT boost for patients with VHR PCa.

## 1. Introduction

Globally, prostate cancer (PCa) is the second most common cancer overall after lung cancer, and, as of 2020, the fifth leading cause of cancer-related deaths in men. It is estimated that in 2020 there were 1.4 million new cases of PCa and 375,000 deaths worldwide [1]. This high incidence is partly attributed to population aging and increased early detection resulting from the widespread use of prostate-specific antigen (PSA) testing [2].

Beyond its elevated incidence and mortality rate, PCa represents a spectrum of diseases for which multiple treatment strategies exist. Proper risk stratification based on defined criteria is crucial in aiding physicians to determine the optimal treatment for each patient. High-risk prostate cancer (HR PCa) patients represent a unique challenge due to the heterogeneity of the group, which encompasses individuals with a broad spectrum of prognoses, some of whom exhibit more aggressive phenotypes that are less responsive to treatment.

To address these challenges, in February 2010, the National Comprehensive Cancer Network (NCCN) issued a significant update that divided patients with HR PCa for recurrence after definitive treatment into two groups: those with high-risk and those with very high-risk prostate cancer (VHR PCa). The 2023 NCCN guidelines [3] currently define patients with VHR PCa as those presenting at least one of the following characteristics: clinical stage T3b-T4, or primary Gleason pattern 5, or more than four grade group 4 or grade group 5 biopsy cores, or having two or three high-risk features. Additionally, several tissue-based prognostic markers have been developed to improve risk stratification in PCa; however, their widespread adoption in routine clinical practice remains limited [4].

Patients with VHR PCa often experience rapid disease progression and have a higher propensity for distant metastases, highlighting the critical need for more effective and intensive treatment modalities to improve outcomes in this particular patient population [5,6,7]. Among the various treatment strategies, dose-escalated radiotherapy has demonstrated superior outcomes in terms of biochemical progression-free survival (bPFS) and local control compared to conventional dose regimens [8,9,10,11,12,13,14,15,16]. The combination of external beam radiation therapy (EBRT) and high-dose-rate interstitial brachytherapy (HDR-BT) as a boost allows for the delivery of high doses of radiation directly to the prostate with minimal exposure of surrounding tissues. This approach increases the biologically effective dose (BED) and enhances tumor control while limiting toxicity [17,18,19,20,21,22,23,24,25].

Aligned with these advances, the 2023 NCCN guidelines [26] recommend, with level A evidence, that VHR PCa patients be treated with EBRT combined with an HDR-BT boost, along with androgen-deprivation therapy (ADT) for two or three years with curative intent. This multimodal approach is predicated on the principle of intensifying local therapy to achieve better local control and potentially eradicate micrometastatic disease, thereby improving long-term survival rates.

Despite these recommendations, evidence for this treatment modality in VHR PCa patients over the long term is limited [27,28,29,30,31,32,33,34]. This gap in knowledge highlights the necessity for robust, long follow-up longitudinal studies to evaluate the sustained efficacy and safety of combined HDR-BT and EBRT in this very high-risk cohort.

This study evaluates the efficacy and safety of an HDR-BT regimen consisting of two fractions of 11.5 Gy, combined with pelvic EBRT administered at a dose of 46 Gy in 23 fractions, in treating patients with VHR PCa. The aim is to determine whether this approach can improve disease control and survival outcomes in this challenging patient population after 24 years of follow-up. By providing long-term data, this research seeks to inform clinical practice and guideline development, ultimately improving care for patients with VHR PCa.

## 2. Materials and Methods

To our knowledge, this is the longest follow-up study in the current medical literature specifically focused on patients with VHR PCa treated with this particular combination of therapies.

### 2.1. Study Design and Patient Population

This retrospective cohort study enrolled a total of 87 patients diagnosed with VHR PCa at our institution between August 1999 and August 2006. Eligibility criteria included patients with histologically confirmed prostate adenocarcinoma meeting the NCCN criteria for very high risk [2]. Specifically, patients had to exhibit at least one of the following criteria: clinical stage T3b-T4, primary Gleason pattern 5, or a combination of ≥2 high-risk features, such as cT3a, Gleason 8–10, or PSA levels >20 ng/mL. Importantly, all patients demonstrated no evidence of nodal or distant metastases and were deemed suitable for treatment and anesthesia during the brachytherapy procedure. Table 1 displays the patient and tumor characteristics.

All cases underwent staging in accordance with the American Joint Committee on Cancer (AJCC) 8th-edition guidelines [35]. Staging procedures included digital rectal examination (DRE), serum PSA measurement, bone scintigraphy, abdominal computed tomography (CT) and/or magnetic resonance imaging (MRI), and transrectal ultrasound (TRUS) biopsy with detailed pathologic reporting. These comprehensive evaluations were crucial for accurately determining the extent of disease and for guiding personalized treatment strategies.

Exclusion criteria were rigorously applied to ensure homogeneity within the study cohort. Patients with a history of prior pelvic radiotherapy or radical prostate surgery, a concurrent or recent (within 5 years) history of another malignancy excluding non-melanoma skin cancer, recurrent PCa, or a projected life expectancy of less than 5 years were excluded from the study.

Written informed consent was obtained from all participants, and the study protocol ensured compliance with both ethical standards and patient confidentiality.

### 2.2. Treatment Protocol

#### 2.2.1. External Beam Radiation Therapy

All patients received EBRT to the prostate, periprostatic tissue, seminal vesicles, and pelvic lymph nodes. The treatment regimen consisted of a total dose of 46 Gy delivered in 23 fractions over 5 weeks. Three-dimensional external beam radiation therapy (3D-EBRT) utilizing 18 MV photons was employed to optimize target coverage while minimizing radiation exposure of adjacent normal tissues.

Dose constraints for the rectum and bladder were as follows: V45 Gy < 15% and V40 Gy < 40%. The small bowel dose constraints were V45 Gy < 64 cc, ensuring no more than 180 cc received 35 Gy. Additionally, the maximum femoral head dose was limited to 46 Gy, with V45 Gy < 25%, and V40 Gy < 40%.

#### 2.2.2. High-Dose-Rate Brachytherapy

HDR-BT was utilized as a boost treatment, delivering a total of 23 Gy in two fractions of 11.5 Gy each, using an Iridium 192 source. This boost was administered to the prostate and medial aspects of the seminal vesicles on days 5 and 15 of the EBRT treatment schedule, with no external radiation therapy given on these days. The total treatment time, including the HDR-BT boost, lasted five weeks.

The HDR-BT procedures were performed under spinal anesthesia, with patients positioned in a forced lithotomy setup for precise catheter placement guided by transrectal ultrasound. The Oncentra Prostate treatment planning system (Nucletron, Elekta, Stockholm, Sweden) facilitated accurate dose distribution and coverage assessment.

The average number of catheters implanted per patient was 15 (range 11–18). While the majority (83 patients) received two implants, technical challenges or specific patient conditions necessitated a single implant in four cases. For these four patients, external beam radiation therapy was escalated to 60 Gy in 30 fractions.

Rectal protection with hyaluronic acid was administered in 26 patients to minimize radiation-induced rectal toxicity. When deemed necessary, cystoscopy was performed at the conclusion of the procedure.

Dose-volume histogram analysis guided treatment planning, ensuring optimal coverage of the planning target volume (PTV) while adhering to strict dose constraints for organs at risk.

The dosimetric criteria for the PTV included the following: D90% ≥ 100% of the prescribed dose, and V90% and V100% ≥ 95% of the prescribed dose. For critical structures, such as the rectum and urethra, the maximum doses were constrained to ≤70% and ≤115% of the prescribed dose, respectively.

HDR-BT procedures were conducted as outpatient treatments, allowing patients to be discharged on the same day, approximately 6–8 h after the implantation.

The total BED resulting from the combination of EBRT and HDR-BT boost ranged from 281 to 366 Gy, calculated using an α/β ratio of 1.2 [36,37].

#### 2.2.3. Androgen-Deprivation Therapy

The incorporation of ADT was based on individualized clinical judgment and patient-specific factors. A significant proportion (82.75%) of patients received neoadjuvant and/or concurrent ADT. Fifty-nine patients received the prescribed hormonal treatment for a minimum duration of 12 months; thirteen patients discontinued treatment prior to completing 12 months, and of these, two were able to tolerate androgen deprivation therapy (ADT) for less than 6 months.

### 2.3. Follow-Up

Patients underwent systematic follow-up assessments to monitor treatment response and detect adverse events. Follow-up visits were scheduled at three-month intervals during the first year after treatment, at six-month intervals during the second and third years, and annually thereafter. Evaluation protocols included regular PSA testing, DRE, MRI, and TRUS biopsy as clinically indicated. Biochemical failure was defined using the Phoenix criteria (PSA nadir + 2 ng/mL), ensuring the standardized assessment of treatment outcomes [38].

Symptom evaluation utilized the International Prostate Symptom Score (IPSS), while the International Index of Erectile Function (IIEF) scale was employed for assessing sexual function. Toxicity profiles were systematically documented using the Common Terminology Criteria for Adverse Events (CTCAE) version 5.0, ensuring comprehensive evaluation of treatment-related side effects.

### 2.4. Statistical Analysis

Cumulative incidence was used to estimate biochemical, local, and distant failure and genitourinary (GU)/gastrointestinal (GI) toxicity. Survival outcomes, including cancer-specific survival (CSS), tumor-free survival (TFS), and overall survival (OS), were analyzed using Kaplan–Meier methods.

Multivariate analysis was performed using the Cox Proportional Hazards Model to assess independent prognostic factors influencing treatment outcomes.

## 3. Results

### 3.1. Oncological Outcomes

With a median follow-up of 188 months (range, 65–285), 26% (23 patients) exhibited biochemical recurrence, 25% (22 patients) experienced clinical relapse, 15% (13 patients) died from PCa, and 60 patients died from other causes. Among the 23 patients with biochemical failure, the median time to PSA failure was 64.9 months (range, 24–204), with 10.3% failing within ≤3 years and 18.4% within ≤5 years. In patients without biochemical failure, the mean (median; range) post-treatment PSA level was 0.22 (0.10; 0–1.9) ng/mL. At the last follow-up, PSA levels were ≤0.2 ng/mL in 90.9%, ≤0.4 ng/mL in 92.4%, <1 ng/mL in 93.9%, and 1–1.9 ng/mL in 6%. Metastatic disease developed in 20 patients.

**Figure 1:**
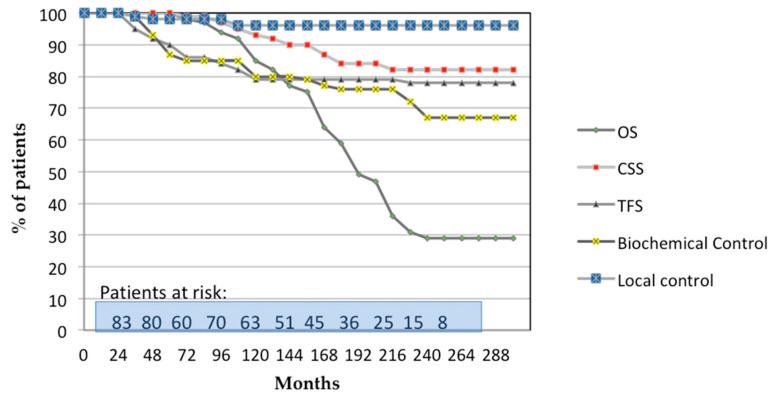
Actuarial analysis of all 87 patients for overall survival (OS), cancer-specific survival (CSS), tumor-free survival (TFS), biochemical control, and local control. Patients at risk is the number of patients in accordance with the months of follow-up.

Local control rates remained at 98% at both 15 and 24 years. Biochemical control rates at 15 and 24 years were 77% and 68% (standard deviation [SD]: ±4%), respectively, while TFS was 79% and 78% (SD ±4%), respectively. CSS at 15 and 24 years was 84% and 82% (SD ±4%), respectively. Based on Kaplan–Meier estimates, the OS was 60% at 15 years and 30% at 24 years (Figure 1).

The factors incorporated into the multiple regression analyses to assess their association with biochemical failure included clinical T-stage, Gleason score, pretreatment PSA levels, primary Gleason pattern 5, brachytherapy dose, prostate volume, year of implantation, use of hormonal ablation therapy (received or not), and age.

**Figure 2:**
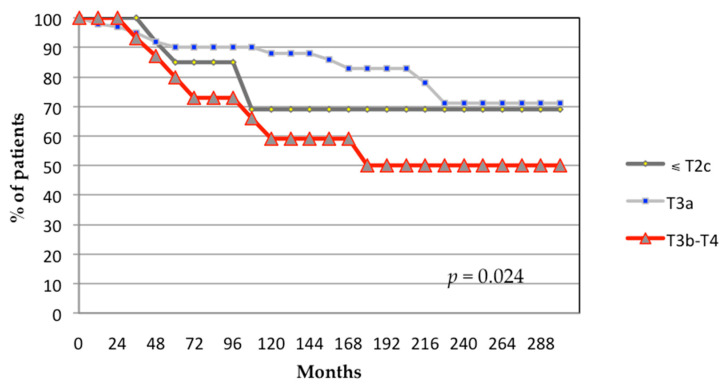
Actuarial analysis of biochemical control based on the T stage in the TNM classification. *p*-value generated from log-rank test.

**Figure 3:**
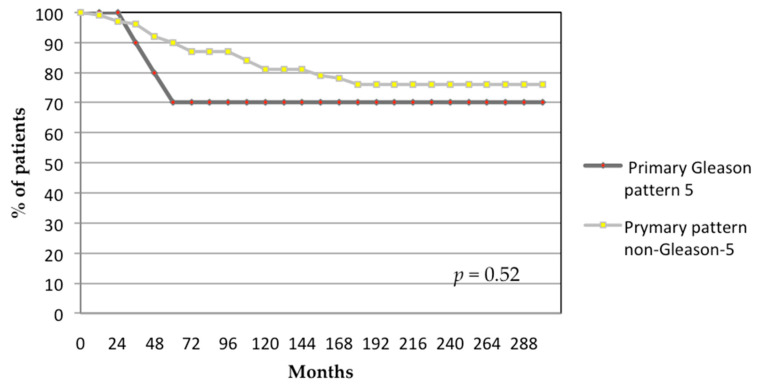
Actuarial analysis of biochemical control by Gleason pattern. *p*-value generated from log-rank test.

Multivariate Cox regression analysis identified only T-classification as an independent prognostic factor for biochemical failure. The 24-year biochemical control rates were 69%, 71%, and 50% for patients with T-classifications of ≤T2c, T3a, and T3b-T4, respectively (*p* = 0.024; Figure 2). Primary Gleason pattern 5 showed no statistically significant differences for biochemical failure (*p* = 0.52; Figure 3).

**Figure 4:**
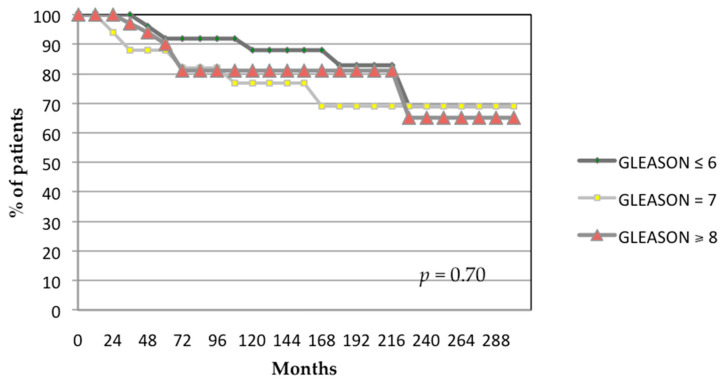
Actuarial analysis of biochemical control by Gleason score. *p*-value generated from log-rank test.

**Figure 5:**
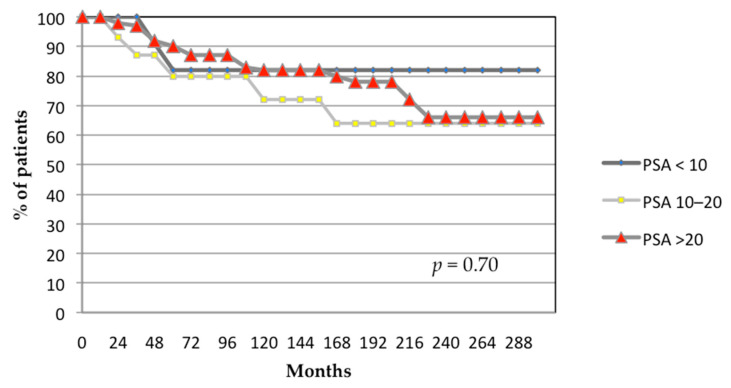
Actuarial analysis of biochemical control by pretreatment PSA (ng/mL). *p*-value generated from log-rank test.

The 24-year actuarial biochemical control rates stratified by Gleason score were 70%, 70%, and 65% for patients with Gleason scores of ≤6, 7, and ≥8, respectively (*p* = 0.70; Figure 4). The 24-year actuarial biochemical control was 82% for patients with pretreatment PSA level < 10 ng/mL, 64% for patients with pretreatment PSA level 10–20 ng/mL, and 66% for patients with pretreatment PSA level > 20 ng/mL (*p* = 0.70; Figure 5).

All other variables, including brachytherapy dose (*p* = 0.93), prostate volume (*p* = 0.70), year of implantation (*p* = 0.51), use of hormonal ablation therapy (*p* = 0.80), and age (*p* = 0.21), were found to be non-significant in relation to biochemical failure.

#### 3.1.1. Toxicity

##### Acute Toxicity

Acute GU toxicity was observed in 17% of patients, predominantly grade 1–2. Common symptoms included dysuria, frequency, and urgency. No cases of acute GU toxicity grade ≥ 3 were observed. Only one patient required catheterization for acute urinary retention, which was then resolved after a few days.

Acute GI toxicity occurred in 9% of patients, predominantly grade 1–2 symptoms such as diarrhea and rectal urgency. Eleven patients (12.6%) experienced rectal bleeding, with only one patient having episodes 1–2 times per week. No grade 3–4 GI toxicities were reported.

##### Late Toxicity

Late GU toxicity was reported in 8% of patients with no grade ≥ 3 toxicity. Late GU toxicities included urinary incontinence, hematuria, and stenosis. No patient experienced incontinence post-treatment. Only one patient required catheterization and dilation for late stenosis.

Late GI toxicity was rare, with only 4.6% of patients experiencing grade 1 toxicity (e.g., mild proctitis). One patient suffered long-term grade 2 GI toxicity (more than four bowel movements per day), and no grade 3–4 events were reported.

##### Sexual Toxicity

Out of the total cohort, 37 patients did not experience erectile dysfunction (ED). Of the remaining patients, 31 presented with grade 1 ED, characterized by a decrease in erectile function (frequency/rigidity of erections) but without the need for therapeutic intervention, while 19 patients were classified as grade 2, requiring medical intervention to address erectile dysfunction. Notably, 12 of these 19 patients (63%) were undergoing ADT, highlighting the contribution of hormone therapy to sexual dysfunction. No cases of grade 3 erectile dysfunction were observed in the study.

## 4. Discussion

Within the classification of HR PCa patients, specific subgroups exhibit a heightened potential mortality rate, necessitating distinct management approaches. Pompe et al. [39] observed a higher incidence of adverse pathologic features and significantly worse oncological outcomes in a cohort of 4000 patients meeting the NCCN criteria for VHR PCa who underwent radical prostatectomy (RP). These outcomes included lower biochemical recurrence-free survival at 5 and 8 years, lower OS, and lower prostate CSS compared to patients with high-risk disease.

Given these findings, the primary challenge for clinicians managing VHR PCa cancer patients is the development of aggressive local treatment strategies that when combined with ADT and/or systemic agents, enable effective and lasting disease control [40].

A promising therapeutic approach that allows for dose escalation within the prostate without increasing toxicity to adjacent organs is the use of an HDR-BT boost combined with EBRT. Utilizing intensity-modulated HDR-BT permits the delivery of a significantly higher BED to the prostate gland that is not currently achievable with intensity-modulated radiation therapy (IMRT) and image-guided radiation therapy (IGRT) techniques and leads to more effective treatment outcomes.

While several trials have evaluated this treatment combination (EBRT+ HDR-BT boost) in HR PCa patients [41,42,43,44,45,46,47], data specific to VHR localized PCa with long-term follow-up (>12 years) remain limited. This study represents a single-institution experience with the longest follow-up to date for VHR PCa patients treated with the EBRT and HDR-BT boost combination. Our findings confirm that this combined therapy yields promising outcomes at 24 years, with a biochemical control rate of 68% and a cancer-specific survival rate of 82%.

It is important to note that despite variability in dosage, fractionation, brachytherapy techniques, and ADT protocols, our results align with those reported by others using HDR-BT as a boost. Hoskin et al. [44], for example, reported a relapse-free survival rate of 71% and 48% at 6 and 12 years, respectively, using a regimen of 55 Gy in 20 fractions followed by two implants of 8.5 Gy each of HDR-BT. Similarly, Tharmalingam et al. [46] reported a 5-year bPFS of 84% in HR PCa patients treated with EBRT to the whole pelvis (46 Gy in 23 fractions) and a single fraction of HDR-BT of 15 Gy. Yamazaki et al. [30] reported a 5- and 10-year bPFS of 81.2% and 71.3%, respectively, in T3b PCa patients and 68.6% and 34.3%, respectively, in T4 tumors. These patients were treated with an EBRT regimen similar to ours but with lower HDR-BT doses (20 Gy in two fractions). Åström et al. [47] reported 10-year biochemical relapse and CSS rates of 65% and 75%, respectively, in VHR PCa patients treated with 50 Gy in 25 fractions and two fractions of 10 Gy of HDR-BT.

As outlined in the ASCO/ASTRO guidelines [48], dose escalation via brachytherapy can be effectively achieved in eligible HR PCa patients through the addition of low-dose-rate brachytherapy (LDR-BT). The ASCENDE-RT trial [49,50] evaluated patients with unfavorable-risk PCa treated with 12 months of ADT, pelvic irradiation to 46 Gy in 23 fractions followed by an Iodine-125 brachytherapy implant with a minimal peripheral dose of 115 Gy. After a median follow-up of 6.5 years, 5-, 7-, and 9-year bPFS rates were 89%, 86%, and 83%, respectively. However, despite these encouraging results with LDR-BT, HDR-BT offers several advantages, including the absence of seed migration or loss, improved dose distribution due to personalized dwell times and inverse planning, the possibility of implanting catheters beyond the prostate, and reduced impact from intraprostatic calcifications [51,52,53]. Additionally, LDR-BT boost has been associated with higher rates of late toxicity. For instance, Rodda et al. [54] reported grade 3 urinary and GI toxicity of 18.4% and 8.1%, respectively, similar to the 15% rate of 3 or higher late toxicity observed by Lawton et al. [55]. In contrast, our study, with a 24-year follow-up, revealed no grade 3 or higher GU or GI toxicities, underscoring the favorable toxicity profile of this treatment regimen. These findings are consistent with other trials that report low rates of grade 3-4 late toxicities (≤ 5%) associated with HDR-BT boost treatment [56,57,58].

Beyond toxicity outcomes, several studies suggest a potential association between radiotherapy for prostate cancer and an increased risk of secondary malignancies, particularly in the bladder, rectum, and colorectal regions [59]. In our cohort, we identified only one case of colon cancer and one case of rectal cancer, with no cases of bladder cancer. The low incidence of these events, combined with other potential confounding factors, makes it difficult to establish a direct causal link to the treatment. Therefore, although these events were documented, we have not emphasized them in the main analysis, as the available data do not support a clear association with radiotherapy.

When comparing this combined-modality treatment with surgical management, oncological outcomes remain contentious. Grimm et al. [60] reported superior progression-free survival rates with combined EBRT and brachytherapy (BT), with or without ADT, in HR PCa patients compared to localized therapies such as RP, BT, or EBRT alone. Furthermore, Kishan et al. [31] found significantly lower cancer-specific mortality in Gleason 9–10 patients treated with combined EBRT and BT compared to those undergoing RP (hazard ratio [HR]: 0.38) or EBRT alone (HR 0.41). However, Moris et al. [61], in an international systematic review, concluded that both RP with adjuvant EBRT and EBRT with hormonal therapy or BT are viable options for locally advanced and HR PCa, with no modality demonstrating superiority in survival outcomes. Similarly, Song et al. [32] and Muralidhar et al. [33] reported comparable CSS between VHR PCa patients treated with RP versus EBRT combined with BT. On the other hand, other trials have reported oncological outcomes in support of the RP option [62,63,64]. The ongoing SPCG15 randomized surgical trial aims to compare RP (with or without adjuvant EBRT) versus EBRT combined with ADT in VHR PCa patients, which will provide further insights. However, until the results of ongoing trials are available, current European guidelines [65] recommend that VHR PCa patients be thoroughly informed about all available treatment options and their respective toxicity profiles, as surgery is associated with greater deterioration in urinary and sexual function, while radiotherapy carries a higher risk of GI toxicity [66,67,68].

In the present study, multivariate Cox regression analysis identified only the highest T categories (T3b-T4) as the sole independent factor for biochemical failure. This finding underscores the potential importance of incorporating HDR-BT as a boost to EBRT, particularly in patients with advanced local disease. HDR-BT’s ability to deliver targeted treatment to both the prostate and the seminal vesicles may enhance biochemical control and improve treatment outcomes in this very high-risk cohort.

However, several factors should be considered when interpreting these results. The retrospective, single-institution design of the study may affect its external validity and limit the generalizability of the findings to other clinical settings. Additionally, the absence of biopsy core data in the pathology reports reflects a common limitation of retrospective studies conducted over extended periods, as such information was not routinely documented in earlier pathology reports. This may have introduced misclassification bias, potentially impacting risk stratification and treatment decision-making. Moreover, the lack of IMRT in this cohort, a technique known to improve oncological outcomes and reduce morbidity compared to 3D-EBRT, may have led to an underestimation of our results [69,70]. It is important to note, however, that 3D-EBRT was the standard treatment modality in most institutions approximately 24 years ago. Many historical studies in radiation oncology share this limitation due to the progressive evolution of radiation techniques over time. Prospective studies with larger patient cohorts are needed and would be valuable to confirm and strengthen the findings of the present study and to ensure their relevance in modern clinical contexts.

Despite these considerations, the long-term biochemical control rates and CSS in this cohort remain excellent, and the low incidence of late toxicities highlights the significant curative potential of this treatment protocol in VHR PCa. This study provides unique and extensive long-term data, offering valuable insights into the management of VHR PCa.

## 5. Conclusions

To our knowledge, this is the longest follow-up study on the clinical outcomes of patients with VHR PCa treated with HDR-BT in combination with whole-pelvic EBRT. This therapeutic approach offers excellent long-term oncological control with minimal late significant toxicities.

## Figures and Tables

**Table 1:** Patient and tumor characteristics (n = 87).

Characteristics	Nº Patients (%)
Stage	
≤T2c	13 (14.9%)
T3a	59 (67.8%)
T3b-T4	15 (17.2%)
Gleason score:	
≤6	25 (28.7%)
7	17 (19.5%)
≥8	45 (52%)
Pretreatment PSA level (ng/mL)	
<10	10 (11.5%)
10–20	16 (18.4%)
>20	61 (70.1%)
Mean pretreatment PSA level (ng/mL): 24.7/Median: 24.4 (3.4–59.6)	
Adjuvant hormonal ablation	
Yes	72 (82.8%)
No	15 (17.2%)
Age at diagnosis (years)	
≤60	11 (12.6%)
61–70	45 (51.7%)
>70	31 (35.6%)
Prognostic factors	
One very high-risk criteria	25 (28.7%)
>One very high-risk criteria	4 (4.6%)
≥Two high-risk criteria	64 (73.6%)
Gland Volume (cc): Mean: 31/Median: 28 (9–69)	

## Data Availability

The original contributions presented in this study are included in the article. Further inquiries can be directed to the corresponding author(s).

## Abbreviations

3D-EBRT	Three-dimensional external beam radiation therapy
ADT	Androgen deprivation therapy
AJCC	American Joint Committee on Cancer
BED	Biologically effective dose
bPFS	Biochemical progression-free survival
BT	Brachytherapy
CSS	Cancer-specific survival
CT	Computed tomography
CTCAE	Common terminology criteria for adverse events
DRE	Digital rectal examination
D90%	The dose that covers 90% of PTV volume.
EBRT	External beam radiation therapy
ED	Erectile dysfunction
GI	Gastrointestinal
GU	Genitourinary
HDR-BT	High-dose-rate brachytherapy
HR	Hazard ratio
HR PCa	High-risk prostate cancer
IGRT	Image-guided radiation therapy
IIEF	International index of erectile function
IMRT	Intensity-modulated radiation therapy
IPSS	International prostate symptom score
LDR-BT	Low-dose-rate brachytherapy
MRI	Magnetic resonance imaging
MV	Megavoltage
NCCN	National Comprehensive Cancer Network
OS	Overall survival
PCa	Prostate cancer
PSA	Prostate-specific antigen
PTV	Planning target volume
RP	Radical prostatectomy
SD	Standard deviation
TFS	Tumor-free survival
TRUS	Transrectal ultrasound
VHR PCa	Very high-risk prostate cancer
V40–45 Gy	% or volume (cc) of PTV receiving 40 Gy and 45 Gy, respectively
V90–100%	% of PTV volume receiving 90% and 100% of the prescribed dose, respectively
